# Analysis of miRNAs involved in mouse brain injury upon Coxsackievirus A6 infection

**DOI:** 10.3389/fcimb.2024.1405689

**Published:** 2024-08-22

**Authors:** Yihao Sun, Yilin Hao, Jie Wu, Shasha Qian, Shuo Shen, Yuting Yu

**Affiliations:** ^1^ Department of Biopharmacy, College of Life Science and Technology, Wuhan Polytechnic University, Wuhan, China; ^2^ Viral Vaccine Research Laboratory I, Wuhan Institute of Biological Products Co. Ltd., Wuhan, China

**Keywords:** hand foot and mouth disease (HFMD), Coxsackievirus A6 (CV-A6), miRNA, brain, central nervous system

## Abstract

**Introduction:**

Coxsackievirus A6 (CV-A6) has emerged as the predominant epidemic strain responsible for hand, foot and mouth disease (HFMD). CV-A6 infection can result in severe clinical manifestations, including encephalitis, meningitis, and potentially life-threatening central nervous system disorders. Our previous research findings demonstrated that neonatal mice infected with CV-A6 exhibited limb weakness, paralysis, and ultimately succumbed to death. However, the underlying mechanism of CV-A6-induced nervous system injury remains elusive. Numerous reports have highlighted the pivotal role of miRNAs in various viral infections.

**Methods:**

Separately established infection and control groups of mice were used to create miRNA profiles of the brain tissues before and after CV-A6 transfection, followed by experimental verification, prediction, and analysis of the results.

**Results:**

At 2 days post-infection (dpi), 4 dpi, and 2dpi vs 4dpi, we identified 175, 198 and 78 significantly differentially expressed miRNAs respectively using qRT-PCR for validation purposes. Subsequently, we predicted target genes of these differentially expressed miRNAs and determined their potential targets through GO (Gene Ontology) enrichment analysis and KEGG (Kyoto Encyclopedia of Genes and Genomes) enrichment analysis. Finally, we verified the miRNA-mRNA pairing via double luciferase experiments while confirming functional enrichment of target genes through Western Blotting analyses.

**Discussion:**

The results from this study suggest that transcriptional regulation, neuronal necrosis, pro-inflammatory cytokine release, and antiviral immunity are all implicated in the pathogenesis of central nervous system injury in mice infected with CV-A6. Brain injury resulting from CV-A6 infection may involve multiple pathways, including glial cell activation, neuronal necrosis, synaptic destruction, degenerative diseases of the nervous system. It can even encompass destruction of the blood-brain barrier, leading to central nervous system injury. The dysregulated miRNAs and signaling pathways discovered in this study provide valuable insights for further investigations into the pathogenesis of CV-A6.

## 1 Introduction

Hand, foot and mouth disease (HFMD) is an infectious illness caused by various human enteroviruses (HEVs), primarily affecting infants under the age of 5, with occasional occurrences in adolescents and adults (Chavan et al., 2023). Typical clinical manifestations of HFMD include mild fever, rash, herpetic or maculopapular eruptions on the hands, soles of feet and buttocks, as well as ulcers in the throat and oral mucosa (Lu et al., 2012; Mehta et al., 2023). Differing from the “classic” enteroviruses associated with HFMD, one of the main pathogens known as Coxsackievirus A6 (CV-A6) can also infect adults leading to atypical symptoms such as generalized herpes or blistering reactions on skin and mucous membranes, desquamation, and even conditions like onychomycosis (Osterback et al., 2009; Wei et al., 2011), along with severe central nervous system disorders including aseptic meningitis and brainstem encephalitis (Xu et al., 2017; Gonzalez et al., 2019).

Since the outbreak in 2008 in Finland, reports of outbreaks caused by CVA6 infection have been documented (Luchs et al., 2022; Bian et al., 2015; Lizasoain et al., 2020). In China, there has been an upward trend in outbreaks/epidemics caused by this pathogen since 2013, surpassing EV71 and CVA16 as the predominant strains responsible for hand, foot and mouth disease (HFMD) in numerous regions (Xu et al., 2020; Li et al., 2014). For instance, a study carried out in Xiangyang, China between October 2016 and December 2017 revealed that CV-A6 infections constituted the largest percentage of cases, at 60.26%, followed by A16 (15.05%), A10 (11.60%), A5 (4.57%), A2 (3.64%), and EV-A71 (2.83%). Consequently, CV-A6 was identified as the predominant pathogen during that timeframe (Meng et al., 2020; Guan et al., 2020). In 2018, a survey conducted in Kunming revealed that the epidemiological characteristics of HFMD and remained consistent from the period before the introduction of the EV-A71 vaccine to three years post its implementation: CV-A6 was identified as the primary pathogen at a rate of 62.33%, followed by CV-A10 at 11.64% and CV-A16 at 10.96% respectively (Jiang et al., 2021). Furthermore, in Chengdu China, EV-71 infections nearly disappeared among HFMD cases between 2013-2022 while more than half were attributed to CV-A6 infections (Yang et al., 2023). Changes in serotypes of epidemic HEV strains have also been observed across other countries and regions (Puenpa et al., 2014; Kimmis et al., 2018). Therefore, Coxsackievirus A6 (CV-A6) has emerged as the primary causative agent of HFMD worldwide. A comprehensive understanding of the pathogenic mechanisms underlying CV-A6 infection is essential for the development of diagnostic tools and vaccines, as well as for effective prevention and treatment strategies against epidemics.

MicroRNAs (miRNAs) are a prominent class of small endogenous non-coding RNAs, approximately 20-25nt in length, that exhibit RNA sequence specificity as post-transcriptional regulatory factors (Cai et al., 2009). They play a pivotal role in diverse cellular activities such as cell proliferation, differentiation, development, and programmed cell death. Mounting evidence has demonstrated the crucial involvement of miRNAs in numerous viral infections, with distinct viral families expressing their own specific miRNAs to exert their functions through manipulation of host miRNA expression or direct/indirect regulation of host or viral miRNAs (Huang et al., 2021; Limthongkul et al., 2019; Khongnomnan et al., 2020; Bernier and Sagan, 2018; Engelmann et al., 2018; Ho et al., 2016). MiRNAs play a crucial role in enterovirus infections. For instance, differentially expressed miRNAs have been implicated in the pathogenesis of Coxsackievirus B3 (CV-B3)-induced viral myocarditis (Garmaroudi et al., 2015). EV-A71-induced miR-494-3p directly facilitates EV-A71 replication by modulating the PI3K/Akt signaling pathway through PTEN targeting (Zhao et al., 2018). CV-A16 disrupts the blood-brain barrier to invade the central nervous system (CNS) via downregulation of miR-1303, thereby directly regulating MMP9 and causing damage to the connective complex, ultimately leading to pathological alterations in the CNS (Song et al., 2018). Findings of these studies suggest a direct involvement of dysregulated host miRNA in the pathogenesis of the virus. Further investigations into cell-encoded miRNAs can enhance our comprehension of the host-virus interaction in enterovirus infection and offer novel strategies for antiviral therapy.

In order to elucidate the pathogenic mechanism of CV-A6 virus and identify changes in miRNA expression following infection, this study established the miRNA profile of mouse brain. The potential targets of differentially expressed miRNAs were determined through GO enrichment analysis and KEGG enrichment analysis. Furthermore, the levels of differentially expressed miRNAs, their target genes, and the functional enrichment of these target genes were validated using Quantitative Real-Time PCR, double luciferase experiments, and western blotting. The findings hold immense importance in advancing our understanding of the mechanisms underlying CV-A6 infection, as well as its prevention and treatment.

## 2 Materials and methods

### 2.1 Cells and viruses

Human malignant embryonic rhabdomyoma cells (RD, RRID: CVCL_1649) were cultured in minimal essential medium (MEM, Nissui, Japan) supplemented with 10% newborn bovine serum (NBS, Gibco), 2 mM glutamine, 100 U of penicillin and 100 μg of streptomycin per ml at 37°C under 5% CO_2_ concentration. The CV-A6 strain HN202009 (the Genbank accession number is PP526685) was utilized for mouse infection (Qian et al., 2021). Virus harvests underwent three freeze-thaw cycles, followed by clarification through centrifugation at a speed of 3900× g for a duration of 10 minutes at a temperature of 4°C. Subsequently, the harvested viruses were stored at -80°C. The titers of CV-A6 stocks were determined by 50% of cell culture infective dose (CCID_50_) assay with the method of Reed and Muench (Lei et al., 2021).

### 2.2 Ethics statement and animal model

The Kuming mice used in this study and the experimental procedures were approved by the Animal Ethics Committee of the Wuhan Institute of Biological Products (WIBP) (WIBP-AII 382020003), adhering strictly to the requirements outlined in the Animal Ethics Procedures and Guidelines of the People’s Republic of China (Standardization Administration of China. Laboratory animal guideline for ethical review of animal welfare. 2018. Standardization Administration of China, Beijing, China.). The permission number assigned is SCXK 2021-0026. As described previously (Qian et al., 2021), we established a neonatal mouse model for CV-A6 infection by intracerebral inoculation (i.c.) with a lethal dose of CV-A6 strain (1.7 × 10^7^ CCID50/mouse). Control and CV-A6-infected mice were euthanized at 2 (n=6/group) or/and 4 days post infection (dpi) (n=6/group), respectively. The brain tissues of all mice were either fixed in 4% paraformaldehyde, embedded in paraffin for histopathological analysis, or stored at −80°C for RNA isolation, RNA-seq, and quantitative PCR (qPCR).

### 2.3 Histopathologic and immunohistochemistry analyses

The tissues were separated, fixed, dehydrated, permeabilized and embedded in paraffin before being sliced into 4 µm sections. Following HE staining, the sealed slides were examined under a microscope.

Rabbit anti-CV-A6 whole virus antiserum at a dilution of 1:200 was added for 15 h at 4°C. After primary anti- body incubation, CV-A6 antigen was detected using agoat-anti-rabbit secondary antibody and a DAB peroxidase (HRP) substrate kit (Solarbio, China). After washing with PBS and dehydration, sealed slides were examined under a microscope.

Add rabbit anti-GFAP, IBA-1, and NeuN at a dilution of 1:200 each, and incubate overnight at 4°C. On the following day, add fluorescent secondary antibody (1:200) and incubate at room temperature for 2 hours. Then, mount the slides with DAPI mounting medium and observe under a fluorescence microscope for photography.

Utilizing Aipathwell software and AI deep learning principles, the nuclei of cells are automatically located and the range of cytoplasm is expanded. The number and area of cells with weak, medium, and strong positivity are calculated. In brief, H-SCORE = ∑ (pi × i) = (percentage of weak intensity × 1) + (percentage of moderate intensity × 2) + (percentage of strong intensity × 3) (Maclean et al., 2020; Dogan et al., 2019; Paschalis et al., 2019; Guo et al., 2019).

To determine the total percentage of positivity in the whole stained sample. Each specimen was assigned a score according to the intensity of the nucleic, cytoplasmic and membrane staining (no staining=0; weak staining=1, moderate staining=2 and strong staining=3) and the extent of stained cells (0~5%=0, 5~25%=1, 26~50%=2, 51~75%=3 and 76~100%=4) (Xie et al., 2014; Wong et al., 2019). The final immunoreactivity score was determined by multiplying the intensity score by the score for the extent of stained cells, generating a score that ranged from 0 (the minimum score) to 12 (the maximum score).

### 2.4 Terminal deoxynucleotidyl transferase dUTP nick end labeling staining

Incubate the sections in an oven at 60°C for 2 hours, followed by a 15-minute incubation at room temperature in 4% PFA. Dilute PBS with 3% hydrogen peroxide to quench endogenous peroxidase activity. As per the manufacturer’s instructions, apply the *in situ* cell death detection kit (fluorescein) (11684795910, Roche) to perform TUNEL staining on the sections. Identify tunel-positive cells with a fluorescence microscope and count the apoptotic cells. Each analysis includes brain samples from 6 mice.

### 2.5 RNA extraction and MicroRNA library construction

Total RNA was extracted from mice brains using TRIzol (Total RNA Extractor (Trizol), Sangon Biotech, China). A total amount of 2.5 ng RNA/mice brain was used as input material for the RNA sample preparations. Leveraging the properties of enzymecatalyzed reactions and molecular biology techniques, the 24 mouse brain samples in this project underwent processes including 3’ end adapter ligation (T4 RNA Ligase 1, New England Biolabs, USA), reverse transcription primer hybridization, 5’ end adapter ligation (T4 RNA Ligase 2, New England Biolabs, USA), cDNA single strand synthesis (M-MuLV Reverse Transcriptase, Sangon, China), and library amplification. Following quality control and purification, a library suitable for sequencing on the Illumina platform was ultimately obtained. The Illumina Xten platform was employed for sequencing. The raw sequencing data contains low-quality reads with adapters. To ensure the quality of data analysis, the raw data is filtered and, considering miRNA characteristics, only reads between 17 and 35 nt in length are retained to produce clean data.

### 2.6 Principal component analysis

The miRNA expression levels in the control-2dpi group, control-4dpi group, 2dpi group, and 4dpi group, were subjected to statistical analysis. Quantify the miRNA counts at different stages of maturation, and normalize the counts to CPM (reads count per million) values. Software used: miRDeep (RRID: SCR_010829). Subsequently, perform PCA analysis using variance decomposition with the R vegan package (version 2.5.6).

### 2.7 Hierarchical cluster tree analysis

Utilize the statistical algorithm, Bray Curtis, to compute the distance between samples, which mirrors the disparities in the collective feature distribution among samples. Subsequently, execute hierarchical clustering analysis, develop a hierarchical structure, and derive a hierarchical relationship for visualized analysis. For software, employ R’s vegan package (version 2.5.6) to calculate the distance, and then utilize hclust to form a clustering tree.

### 2.8 MiRNA differential analysis

DESeq2 (RRID: SCR_015687) was employed to analyze the differential expression of miRNAs in mice across different phases (2 dpi, 4 dpi, control-2dpi and control-4dpi group). To identify significantly differentially expressed genes, the screening criteria were set as follows: p-value <= 0.05 and fold change >= 2. Based on these criteria, volcano plots were constructed. Furthermore, heatmaps were generated based on the combined Foldchange, p-Value, and q-Value values of differentially expressed miRNAs in the comparison group.

Based on the reference genome, use mirDeep2 software to predict novel miRNAs.

### 2.9 Target gene prediction

The miranda (RRID: SCR_017496) algorithm is used to predict miRNA target genes. The miranda algorithm integrates two computational prediction steps, from miRNA-3’UTR sequence matching and energy stability evaluation, to predict miRNA target genes. This algorithm uses dynamic programming algorithms to search for regions where the miRNA and 3’ UTR are complementary and form a stable double-strand. The threshold parameters used in the process of predicting miRNA target genes are: S >= 150, Δ G <= -30 kcal/mol and Demandstrict 5’ seed pairing. Where S refers to the single-residue-pair match scores in the matching region; ΔG is the free energy when the double-strand is formed.

### 2.10 Functional enrichment analysis of differentially expressed miRNA target genes

Gene Ontology (GO, http://www.geneontology.org) provides a dynamically updated controlled vocabulary to comprehensively describe the properties of genes and gene products in organisms. Kyoto Encyclopedia of Genes and Genomes (KEGG, http://www.kegg.jp) integrates genome, chemical substance, and system function information, and can complete functional annotation of the genome or transcriptome of newly sequenced species. After selecting target genes, first study the distribution of target genes in the annotated functions, and then use clusterProfiler (RRID: SCR_016884) for functional enrichment analysis. When the corrected P value (Qvalue) is <0.05, it is considered that the function is significantly enriched.

### 2.11 Functional enrichment association analysis of differentially expressed miRNA target genes

Function and gene are not simply a one-to-one relationship, but a complex many-to-many relationship. Through correlation analysis, functional-functional and functional-gene interaction networks are constructed, and network analysis methods are used to identify key functions and genes. Based on regulatory relationships, the mechanisms of gene and function are clarified.

### 2.12 Quantitative real-time PCR

qRT-PCR was employed to monitor the alterations in the CV-A6 viral load in the brain tissues of mice across different groups (2 dpi group, 4 dpi group, control-2dpi and control-4dpi group). Quantitative Real-Time PCR (qRT-PCR) was conducted using the One Step TB Green PrimeScript RT-PCT Kit II (Perfect Real Time) (Takara Biomedical Technology (Beijing) Co., Ltd., China), following the provided protocol. The primers used for detecting CV-A6 VP1 were VP1 F (5′-ATATTCGCAAAATTGAGTGATCCAC), and VP1 R (5′-GTTATTAGGACATTGCCCATATTGC). Based on the significance of miRNA differences, miRNAs with high differential expression were selected from the 2dpi, 4dpi, and 2dpi4dpi groups for qRT-PCR analysis to assess the reliability of RNA-seq. MiRNA 1st Strand cDNA synthesis Kit (by stem-loop) (Vazyme Biotech Co., Ltd, Nanjing, China) was used to synthesize the cDNA, followed by qRT-PCR analysis using miRNA Universal SYBR qPCR Master Mix (Vazyme Biotech Co., Ltd, Nanjing, China). U6 was employed as the reference gene for normalization(Forward primer 5’ to 3’ is CTCGCTTCGGCAGCACA, Reverse primer 5’ to 3’ is AACGCTTCACGAATTTGCGT);. The primers for miRNA amplification used in this analysis are detailed in **Table 1**.

**Table 1:** Primers used in this study.

miRNA	Forward primer (5’ to 3’)	Reverse transcription primer(5’ to 3’)
miR-196a-5p	CGCGCGTAGGTAGTTTCATGTT	GTCGTATCCAGTGCAGGGTCCGAGGTATTCGCACTGGATACGACCCCAAC
miR-10b-3p	CGCGCAGATTCGATTCTAGG	GTCGTATCCAGTGCAGGGTCCGAGGTATTCGCACTGGATACGACTATTCC
miR-205-5p	CGTCCTTCATTCCACCGG	GTCGTATCCAGTGCAGGGTCCGAGGTATTCGCACTGGATACGACCAGACT
novel-130	GCGCGGGGCTGGTGAG	GTCGTATCCAGTGCAGGGTCCGAGGTATTCGCACTGGATACGACAGCCAT
miR-3473b	GCGGGGCTGGAGAGATG	GTCGTATCCAGTGCAGGGTCCGAGGTATTCGCACTGGATACGACCTGAGC
miR-344g-3p	CGCAGGCTCTAGCCAGGG	GTCGTATCCAGTGCAGGGTCCGAGGTATTCGCACTGGATACGACTCAAGC
miR-7080-3p	GCAGGCTCACCCCTCCG	GTCGTATCCAGTGCAGGGTCCGAGGTATTCGCACTGGATACGACAGGGAA
novel-41	GCGCGAGTGAGTTCCAGGA	GTCGTATCCAGTGCAGGGTCCGAGGTATTCGCACTGGATACGACTGGCTG
novel-98	GCGCGCTGGCTGGCCT	GTCGTATCCAGTGCAGGGTCCGAGGTATTCGCACTGGATACGACAGTTCC
miR-3963	CGCGCGTGTATCCCACTTC	GTCGTATCCAGTGCAGGGTCCGAGGTATTCGCACTGGATACGACGTGTCA
novel-1	CGCGCGATCCCACTTCT	GTCGTATCCAGTGCAGGGTCCGAGGTATTCGCACTGGATACGACTGTGTC
novel-151	CGCGCGTGTCTCTCCAGT	GTCGTATCCAGTGCAGGGTCCGAGGTATTCGCACTGGATACGACAAGGTG
novel-73	GCGATAAGGGGTGGAGAGTT	GTCGTATCCAGTGCAGGGTCCGAGGTATTCGCACTGGATACGACTGAGCC
novel-94	CGCGTCCACAGCTGTGACTA	GTCGTATCCAGTGCAGGGTCCGAGGTATTCGCACTGGATACGACAAAATG
miR-205-3p	CGCGGATTTCAGTGGAGTGA	GTCGTATCCAGTGCAGGGTCCGAGGTATTCGCACTGGATACGACTGAGCT
novel-173	GCGCGATGGCACTTTATCAT	GTCGTATCCAGTGCAGGGTCCGAGGTATTCGCACTGGATACGACGTTGCT

Reverse primer: AGTGCAGGGTCCGAGGTATT.

### 2.13 Luciferase reporter assay

Briefly, the 3 ‘- UTR fragments of the target genes Dvl2, Axin2 and their mutants were first cloned into the XhoI and NotI sites of the psiCHECK-2 vector, and the target gene fragment contained predicted miRNA binding sites. In addition, a negative control group for binding site mutations was established. Then the recombinant plasmid was transfected into HEK 293T cells using the Lipofectamine 3000 transfection reagent (Thermo Fisher Scientific Inc. USA). After 48 hours of transfection, the cells were collected and subjected to dual-luciferase reporter assay using the Dual-Luciferase^®^ Reporter Assay System (Promega (Beijing) Biotech Co., Ltd, China) according to the manufacturer’s instructions. The activities were normalized to the Renilla luciferase signal in HEK 293T cells.

### 2.14 Western blotting

Total protein was extracted from the brain tissues of mice in the CV-A6 infection (4 dpi) group and the control-4dpi group using a whole protein extraction kit (Beijing Solarbio Science & Technology Co., Ltd., China). Protein samples were separated by 12% SDS-PAGE, then transferred to PVDF membranes. A blocking solution was applied and incubated overnight at 4°C. After the primary antibodies were incubated at room temperature for 2 hours, the membranes were washed 3 times with PBST (phosphate-buffered saline, pH 7.6, containing 0.05%Tween20), then incubated with rabbit secondary antibodies at room temperature for 1 hour, and then washed 3 times with PBST. Finally, color development and photos were taken. The gray values of the images were determined using Image J (RRID: SCR_003070), and statistical analysis was conducted using GraphPad Prism (RRID: SCR_002798).

### 2.15 Antibodies

The following primary antibodies were used in this study: Rabbit anti-CV-A6, rabbit monoclonal to β-Catenin (Abcam Biotechnology, Inc.), rabbit monoclonal to LEF1(Abcam Biotechnology, Inc.), rabbit monoclonal to Cyclin D1 - C-terminal (Abcam Biotechnology, Inc.), rabbit monoclonal to Axin (Abcam Biotechnology, Inc.), rabbit monoclonal to GSK3β(Abcam Biotechnology, Inc.), anti-PLCG1 (Phospho-Tyr783) rabbit polyclonal antibody (Sangon Biotech (Shanghai) Co., Ltd., Shanghai, China), anti-IL-1 rabbit polyclonal antibody (Sangon Biotech (Shanghai) Co., Ltd., Shanghai, China), anti-IL-6 rabbit polyclonal antibody (Sangon Biotech (Shanghai) Co., Ltd., Shanghai, China), anti-TNF-αrabbit polyclonal antibody (Sangon Biotech (Shanghai) Co., Ltd., Shanghai, China).

### 2.16 Statistical analyses

Statistical analysis was conducted with GraphPad Prism (RRID: SCR_002798). The results were represented as the mean ± standard deviation (SD). One-way analysis of variance (ANOVA) or unpaired Student’s t test was used to determine the significance of differences between groups. Statistical significance was indicated as follows: n.s., not significant (P ≥ 0.05); *, 0.01 ≤ P < 0.05; **, P < 0.01; ***, P < 0.001 and ****, P < 0.0001.

## 3 Results

### 3.1 Mouse model infected with CV-A6

Previously, we had already established a mouse model for CV-A6 infection. The results showed that inoculation via the i.c. route began to show symptoms at 2 dpi and eventually all died within 4 dpi, and 100% of control mice are alive (Qian et al., 2021). The mice in this study exhibited clinical symptoms at 2 dpi, consistent with previous findings, and succumbed to mortality by 4 dpi.

**Figure 1:**
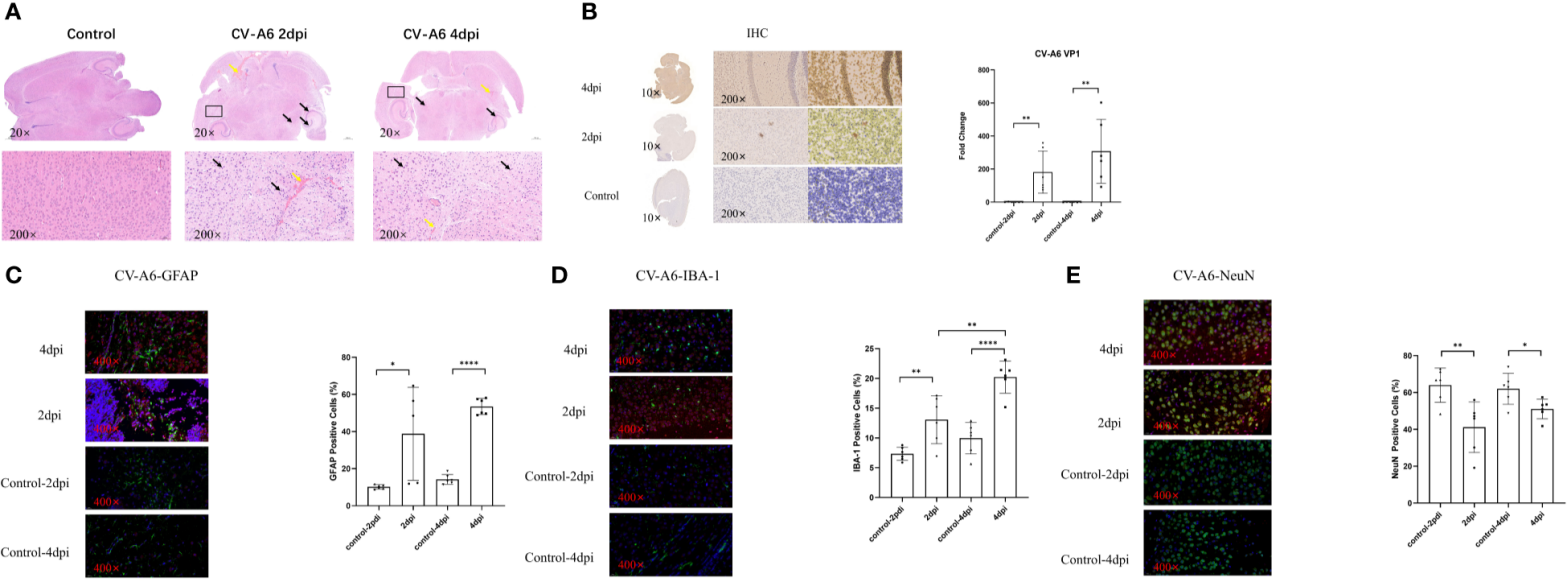
Brain tissues of mice were damaged after CV-A6 infection. **(A)** Histopathological changes in the brain tissue sections of mice were evaluated by H&E staining. The black arrow signifies the shrinkage of neurons, while the yellow arrow marks the site of bleeding. **(B)** Viral proteins in the brain tissue sections of mice were evaluated by immunohistochemical staining. The viral titer was expressed as the fold change of VP1 mRNA in the brain tissues of mice detected by qRT-PCR (n=6). The results were calculated as 2^-△Ct. **(C)** Colocalization of CV-A6 with GFAP in the mouse brain tissue, and the GFAP positive cell rate was determined. **(D)** Colocalization of CV-A6 with IBA-1 in the mouse brain tissue, and the IBA-1 positive cell rate was determined. **(E)** Colocalization of CV-A6 with NeuN in the mouse brain tissue, and the NeuN positive cell rate was determined. Original magnification, × 20, Scale bar = 500 μm; × 200, Scale bar = 50 μm; × 400, Scale bar = 20 μm. Red light represents CV-A6 staining, green light represents GFAP, IBA-1, and NeuN staining in different figures, and blue light represents DAPI staining. *p < 0.05; **p < 0.01; ***p < 0.001; **** p<0.00001.

The experimental results showed that significant detection of CV-A6 VP1 in the brain tissues of mice at both 2dpi and 4dpi. There were varying degrees of pathological changes in the brain tissues of mice within 2 and 4 days after infection, a small number or many neurons in the cortex, striatum, CA1, CA2, CA3 regions of the hippocampus, thalamus, and striatum showed shrinkage, with cells stained deeper and nuclei and cytoplasm demarcated unclearly, and multiple hemorrhages were visible (**Figure 1A**). Immunohistochemical analysis demonstrated a substantial presence of CV-A6 virus particles in the brain tissues of mice at both 2dpi and 4 dpi, with a noticeable upward trend in the 4 dpi samples compared to the 2 dpi samples, as indicated by H-SCORE values of 199.7 ± 11.75 and 111.9 ± 10.594, respectively (**Figure 1B**). The positive cell rates of GFAP and IBA-1 in the 2dpi and 4dpi groups after infection significantly increased compared to their respective control groups (**Figures 1C, D**). Compared to the control group, the positive cell rates of NeuN in the 2dpi and 4dpi mice significantly decreased (**Figure 1E**). Based on the preceding experimental results, it can be concluded that the CV-A6 virus continues to proliferate in the brain tissues of newborn mice, leading to severe pathological damage.

### 3.2 MiRNA profiling of the brain tissue from CV-A6 infected mice

**Figure 2:**
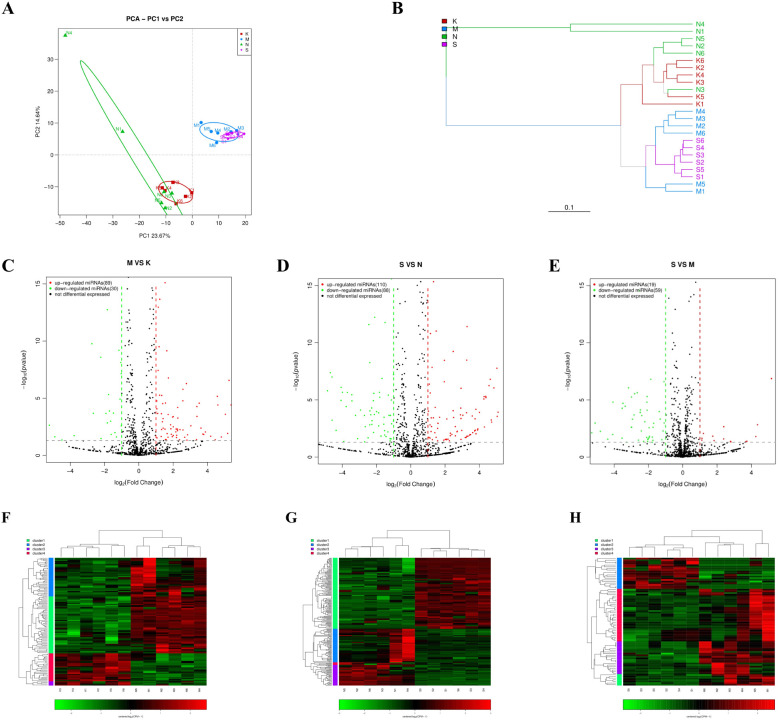
The miRNA expression pattern in brain tissue of mice was analyzed after CV-A6 infection. **(A)** Principal component analysis (PCA) plot was used to visualize the sample groups under different environments or conditions, with dots of different colors or shapes representing each group. The scales of the horizontal and vertical coordinates are relative distances without practical significance. **(B)** Hierarchical clustering tree analysis was performed to assess the similarity between samples, with branch lengths indicating the distance between samples. Samples were distinguished by different colors based on grouping. **(C–E)** An expression difference volcano plot was generated to illustrate the fold-change (log (B/A)) value of gene expression differences between sample groups on the horizontal axis, and the statistical significance of gene expression changes (pValue) on the vertical axis. Smaller pValues corresponded to larger -log(pValue), indicating greater significance in differential gene expression. Each dot represented a gene, where red indicated up-regulated genes, green indicated down-regulated genes, and black indicated non-differential genes. **(F–H)** Heatmap of differentially expressed miRNAs. Each row represents a gene and each column represents a sample. The higher the color is, the higher the expression level is. S: mice in the 4dpi group, M: mice in the 2dpi group, N: control-4dpi group, K: control-2dpi group.

The miRNA expression profiles of mice were analyzed in the 2 dpi, 4 dpi, control-2dpi and control-4dpi group. MiRNA sequences specific to the species were retrieved from the miRbase (https://www.girinst.org/repbase/) database and identified using mirDeep2[4] (version 2.0.0.8) software. A total of 889 known miRNAs and 190 novel miRNAs were identified. In the PCA principal component analysis, miRNAs from infected mice and control mice formed independent clusters (**Figure 2A**). This expression pattern was also reflected in the hierarchical clustering tree (**Figure 2B**). The findings revealed a stark contrast in miRNA expression between post-infection mice and control mice. Yet, there were no distinct clusters in miRNA expression between 2dpi group and 4dpi group, nor between control-2dpi and control-4dpi group, indicating minimal differences.

Through miRNA differential analysis, 943, 1026, and 1028 miRNAs, including known and novel miRNAs, were identified in the 2dpi vs control-2dpi, the 4dpi vs control-4dpi, and the 4dpi vs 2dpi, respectively. Among them, 175, 198, and 78 miRNAs demonstrated significant differential expression (p-value <= 0.05, fold change ≥ 1) at 2dpi vs control-2dpi, 4dpi vs control-4dpi, and 4dpi vs 2dpi. Specifically, 129 miRNAs were consistently differentially expressed in both the 2dpi vs control-2dpi and 4dpi vs control-4di, with 128 showing the same increase/decrease trend; whereas 16 miRNAs were consistently differentially expressed across the three comparison strategies, with 11 showing the same increase/decrease trend (**Supplementary Table S1**). The volcano plot (**Figures 2C–E**) and heatmaps (**Figures 2F–H**) visually represents the changes in miRNA expression between groups. This finding provides insights into the altered landscape of mouse brain tissue’s miRNA caused by CV-A6.

### 3.3 Functional enrichment analysis of differential miRNA target genes

**Figure 3:**
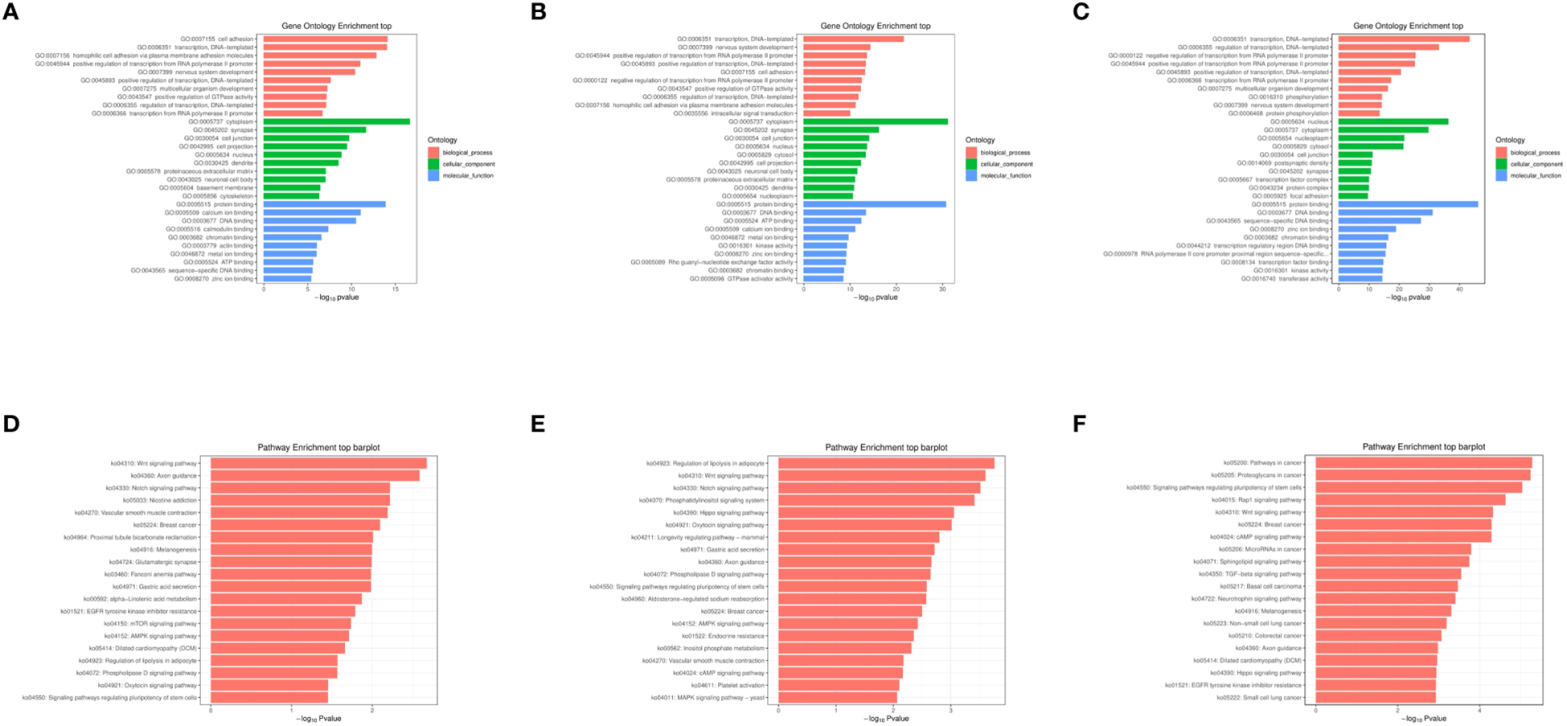
GO and KEGG enrichment analysis of miRNAs exhibiting significantly aberrant expression in mouse brain tissues following CV-A6 infection were performed. **(A)** GO enrichment analysis was conducted on miRNAs with significantly abnormal expression in the brain tissues of mice at 2dpi vs control-2dpi. **(B)** GO enrichment analysis was carried out on miRNAs with significantly abnormal expression in the brain tissues of mice at 4dpi vs control-2dpi. **(C)** GO enrichment analysis was carried out on miRNAs with significantly abnormal expression in the brain tissues of mice at 4dpi vs 2dpi. **(D)** KEGG enrichment analysis was performed on miRNAs with significantly abnormal expression in the brain tissues of mice at 2dpi vs control-2dpi. **(E)** KEGG enrichment analysis was executed on miRNAs with significantly abnormal expression in the brain tissues of mice at 4dpi vs control-4dpi. **(F)** KEGG enrichment analysis was executed on miRNAs with significantly abnormal expression in the brain tissues of mice at 4dpi vs 2dpi.

The objective of conducting functional enrichment analysis on differentially expressed miRNA target genes is to identify whether these genes are enriched in specific biological functions. GO and KEGG enrichment analyses were performed separately for the differentially expressed miRNAs between 2dpi, 4dpi, control-2dpi and control-4dpi mice groups. The GO enrichment analysis covered three domains: biological process (BP), cell component (CC), and molecular function (MF). Bar graphs were generated for each domain, representing the results for 2dpi vs control-2dpi, 4dpi vs control-4dpi, and 4dpi vs 2dpi (**Figures 3A–C**). Based on the p-value results, the prominent GO terms included transcription, DNA−templated (BP), nervous system development (BP), positive regulation of transcription, DNA−templated (BP), positive regulation of transcription from RNA polymerase II promoter (BP), cell adhesion (BP), synapse (CC), cell junction (CC), cell projection (CC), protein binding (MF), ATP binding (MF), DNA binding (MF), calcium ion binding (MF). KEGG pathway enrichment analysis was employed to determine the biological pathways and functions associated with differentially expressed miRNAs in CV-A6-infected mice at 2dpi vs control-2dpi, 4dpi vs control-4dpi, and 4dpi vs 2dpi (**Figures 3D–F**). According to the p-value results, the main pathways identified included: Wnt signaling pathway, Notch signaling pathway, and phosphatidylinositol signaling pathway.

### 3.4 Functional enrichment and correlation analysis of differentially expressed miRNA target genes

**Figure 4:**
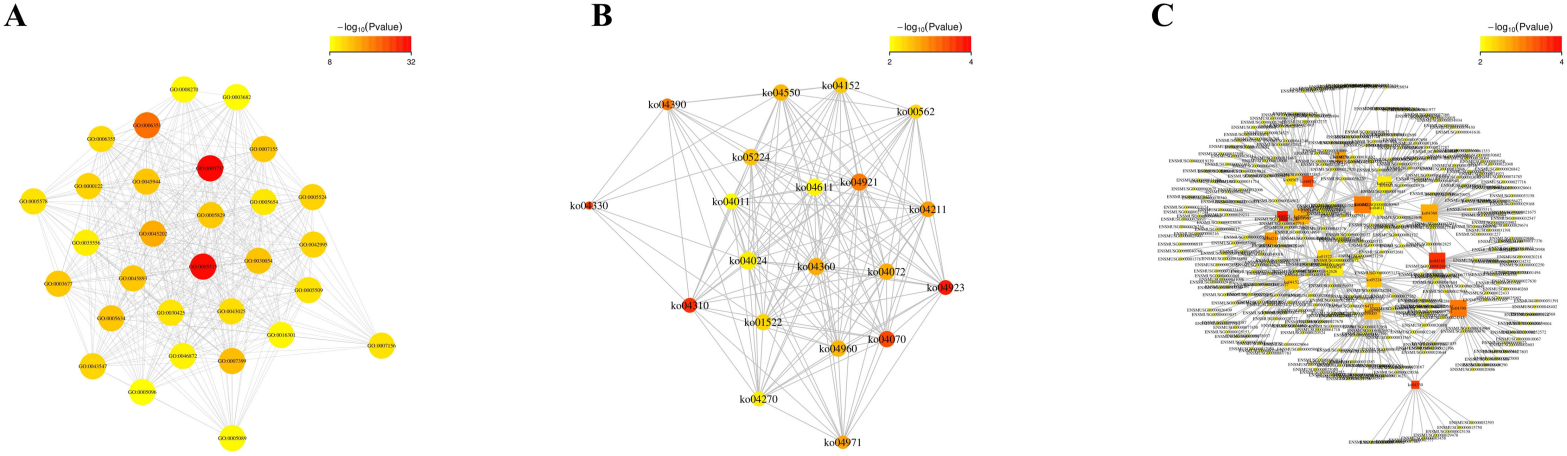
Functional enrichment correlation analysis was performed on the differentially expressed miRNA target genes in mouse brain tissues following CV-A6 infection. **(A)** The significantly enriched function-function interaction network diagram was presented, where circular nodes represented functional information and edges represented the correlation between functions. The color of nodes indicated the degree of function enrichment, namely P-value value. Nodes with higher degrees of enrichment had lower P-values and appeared red. (Only the top 50 functions with the highest degree of enrichment were included.) **(B)** Another significantly enriched function-function interaction network diagram was shown, using circular nodes to represent functional information and edges to represent correlations between functions. The color of nodes reflected the degree of function enrichment, namely P-value value. Nodes with higher degrees of enrichment had lower P-values and appeared red. **(C)** Additionally, a significantly enriched function-gene interaction network diagram was displayed, where square nodes represented functional information, circular nodes represented genes, and edges depicted correlations between genes and functions. The size of each node corresponded to its connectivity or degree; larger nodes indicated more connections.

The relationship between function and gene is characterized by intricate many-to-many connections. By conducting association analysis, we investigated the functional enrichment association between 4dpi and the target genes of differentially expressed miRNAs in the control-4dpi group. Additionally, we constructed an interaction network to identify key functions and genes involved in these associations (**Figures 4A–C**). The nodes in the network mainly include GO-GO: protein binding, cytoplasm, transcription, DNA-templated; KO-KO (pathway-pathway): Wnt signaling pathway, Phosphatidylinositol signaling system, Regulation of lipolysis in adipocyte; function-gene: Wnt signaling pathway (45 genes), Oxytocin signaling pathway (47 genes), Hippo signaling pathway (46 genes).

### 3.5 Validation of differentially expressed miRNAs by quantitative real-time PCR

**Figure 5:**
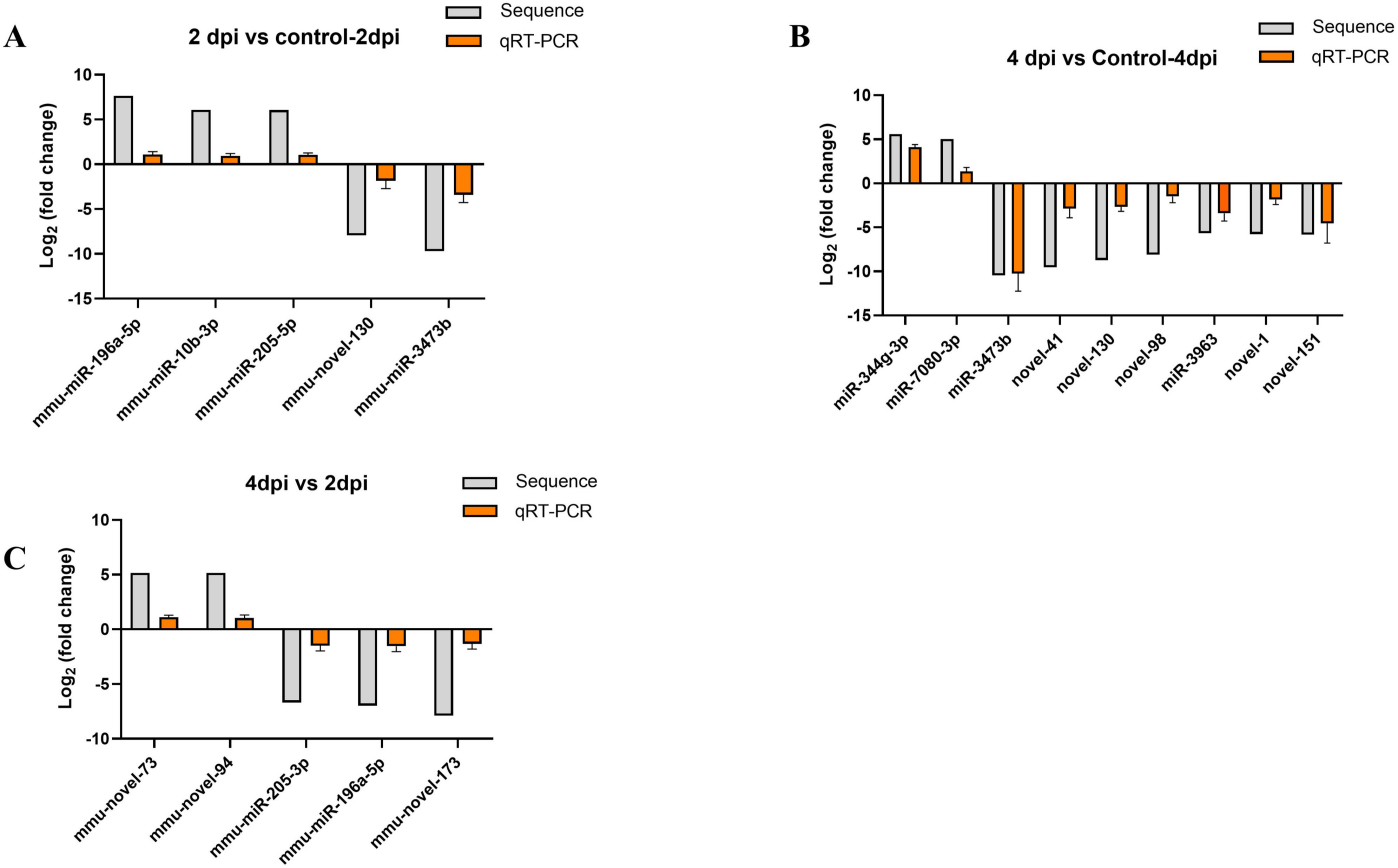
Verification of RNA-seq using qPCR. **(A)** The 5 miRNAs changed at 2dpi vs control-2dpi. **(B)** The 9 miRNAs changed at 4dpi vs control-4dpi. **(C)** The 5 miRNAs changed at 4dpi vs 2dpi.The detection for each miRNA was repeated at least 3 times and the standard deviation was.

The reliability of differentially expressed miRNAs, identified by sequencing, was confirmed through qRT-PCR analysis. Based on the sequencing results, 5, 9, and 5 miRNAs exhibiting the most significant expression differences were chosen for analysis from the comparisons 2dpi vs control-2dpi, 4dpi vs control-4dpi, and 4dpi vs 2dpi, respectively. We utilized control mice as a normalization strategy to assess the fold change of differentially expressed miRNAs in the brain tissue of CV-A6 infected mice. Based on the log2FoldChange calculations, some subsets of significant differences miRNAs exhibited either upregulation or downregulation following infection in the mouse brain tissue, which was consistent with the sequencing results. (**Figures 5A–C**).

### 3.6 Validation of miRNA target genes

**Figure 6:**
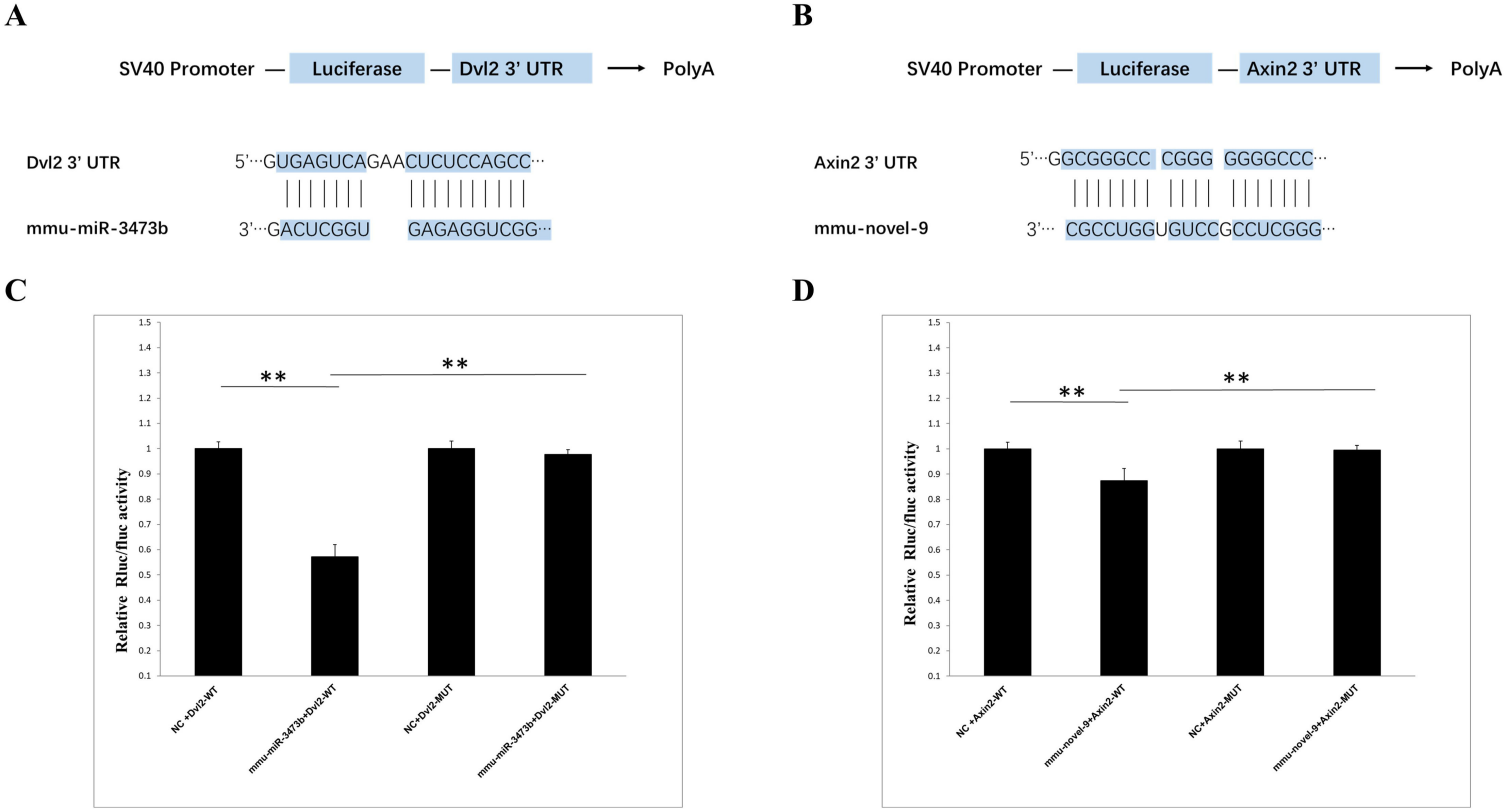
Validation of miRNA and target gene. **(A)** Schematic representation of the binding sites between mmu-miR-3473b and Dvl2. **(B)** Schematic illustration of the binding sites between mmu- novel-9 and Axin2. **(C)** Observed changes in reporter gene expression comparing Dvl2-WT and Dvl2-MUT groups. **(D)** Observed changes in reporter gene expression comparing Axin2-WT and Axin2-MUT groups. Luciferase reporter gene assay was conducted using 293T cells with firefly luciferase serving as an endogenous control. Data are presented as mean ± SD from three independent experiments (**p < 0.01).

The primary targets of animal miRNAs are the 3’ untranslated region (3’ UTR) of mRNAs, through which they exert their functional mechanisms by suppressing translation. Based on the sequencing report, two miRNA-target gene pairs exhibiting significant differences between the 4dpi and control-4dpi groups were chosen for experimental validation. The genes Dvl2 (Wnt signaling pathway) and Axin2 (Wnt signaling pathway) were hypothesized to be potential targets of mmu-miR-3473b and mmu-novel-9. The reliability of the sequencing data was confirmed through a double luciferase experiment. The Targetscan database (http://www.targetscan.org/vert_72/) was utilized for prediction, revealing the presence of binding sites between Dvl2 and Axin2 with mmu-miR-3473b and mmu-novel-9 respectively (**Figures 6A, B**). The luciferase experiments demonstrated that mmu-miR-3473b effectively modulated the expression of the luciferase reporter gene upon co-transfection with Dvl2 and Dvl2 (MUT) into 293T cells. Furthermore, restoration of normal reporter gene expression was observed upon mutation of the binding site (Dvl2 (MUT)). Similarly, consistent results were obtained for mmu-novel-9 and Axin2 as well as Axin1 (MUT) (**Figures 6C, D**). These findings provide evidence for specific interactions between mmu-miR-3473b and Dvl2, as well as mmu-novel-9 and Axin2, confirming the accuracy of the identified binding sites.

### 3.7 Western blot verification of functional enrichment of miRNA target genes and cytokine expression levels

**Figure 7:**
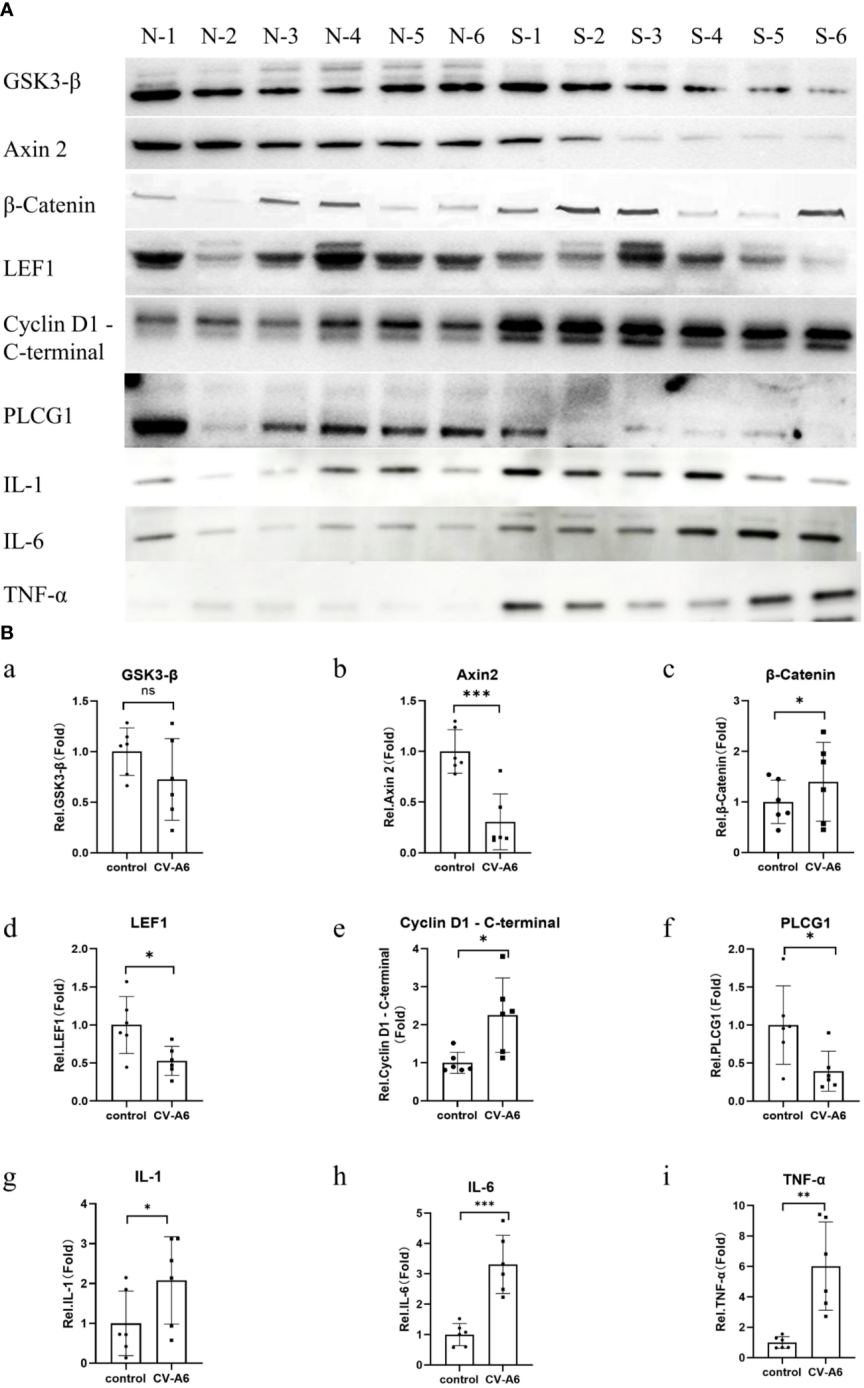
Validation of functional enrichment. Brain tissues were obtained from 4dpi (n = 6) and control-4dpi group (n = 6) and subjected to Western blotting (n = 6). **(A)** Western blot analysis of Axin 2, GSK3-β, β-Catenin, LEF1, Cyclin D1 - C-terminal, PLCG1, IL-1, IL-6, and TNF-α. **(B)** Expression levels of Axin 2, GSK3-β, β-Catenin, LEF1, Cyclin D1 - C-terminal, PLCG1, IL-1, IL-6, and TNF-α. *p < 0.05, **p < 0.01, ***p < 0.001.

To verify the reliability of GO enrichment analysis and KEGG enrichment analysis results, Western Blot was performed on Wnt signaling pathway (Axin 2, GSK3-β, β-Catenin, LEF1, Cyclin D1 - C-terminal protein), and Phosphatidylinositol signaling system (PLCG1). Additionally, the levels of IL-1, IL-6, and TNF-α were further assayed using anti-IL-1, anti-IL-6, and anti-TNF-alfa antibodies. The findings revealed that, following CV-A6 infection, the expression levels of Axin 2, LEF1 and PLCG1 proteins in the mouse brain significantly diminished, whereas those of β-Catenin and Cyclin D1 - C-terminal proteins markedly rose (**Figures 7A, B**). This suggests that the Wnt signaling pathway is upregulated in the brain tissue of mice infected with CV-A6. Concurrently, the levels of IL-1, IL-6, and TNF-α in the brain tissues of infected mice increased (**Figures 7A, B**). These are pro-inflammatory cytokines, indicating an inflammatory response in the mouse brain tissues.

## 4 Discussion

The infection of CV-A6 can lead to severe clinical symptoms, such as encephalitis and meningitis, and even life-threatening diseases of the central nervous system (Hussain et al., 2023; Li et al., 2020; Yang et al., 2020; Xu et al., 2020). The findings from our previous investigations have demonstrated that neonatal mice infected with CV-A6 experience limb weakness, paralysis, and ultimately succumb to mortality (Qian et al., 2021). Additionally, previous studies have demonstrated that CV-A6 mouse models display neurotropism and elicit systemic symptoms (Sun et al., 2023; Jiang et al., 2021a; Zhang et al., 2017; Li et al., 2022). The precise pathophysiological mechanisms underlying nervous system injury resulting from CV-A6 infection remain incompletely understood. Therefore, this study aims to investigate the role of miRNA in neonatal mice with CV-A6 infection and analyze the alterations in miRNA expression within their brain tissues.

Compared with the respective control groups, the positive cell rates of GFAP and IBA-1 in the mouse brain tissues significantly increased, and the levels of IL-1β, IL-6, and TNF-a significantly increased in 2dpi and 4dpi groups.Iba-1 and GFAP are used as molecular markers for microglia and astrocytes, respectively, and their levels indicate the activation state of these cells. Activated microglia release pro-inflammatory cytokines, including IL-1β, IL-6, and TNF-a (Alyu and Miriş, 2017; Tang and Le, 2016).The rate of NeuN-positive cells significantly decreased. NeuN is a specific marker of the nucleus of mature neuronal cells, and a decrease in NeuN staining means that there is neuronal dysfunction, such as antigenic changes, pro-inflammatory cytokine release, synaptic dysfunction, and impaired chemical reception (Unal-Cevik et al., 2004), which are basic mechanisms for maintaining life functions (Feldman et al., 2003). In some pathological conditions, such as cerebral ischemia, hypoxia, and traumatic neurons, the NeuN immunoreactivity decreases (Fang et al., 2024; Igarashi et al., 2001; Xu et al., 2002). The mechanism of CV-A6 infection may be that the virus triggers the activation of glial cells in the mouse brain by damaging neurons and inducing infection-related stimuli (Okuma et al., 2014). Activated microglia and injury neuron release pro-inflammatory cytokines, which can interact with vascular endothelial cells to disrupt the blood-brain barrier, and then act on neurons again, thereby aggravating brain damage and neurological injury.

In the significantly differential miRNA expression spectrum, multiple miRNAs displayed common up-regulation/down-regulation patterns in both 2dpi and 4dpi samples. However, principal component analysis did not reveal distinct clusters formed by differentially expressed miRNAs in these time points. These results indicate significant differences in miRNA expression during the early stage of mouse disease progression, with further changes observed as the infection worsens.

The 2dpi/4dpi mice in this study exhibited a consistent alteration pattern for the aforementioned miRNA, suggesting that CV-A6 infection exerts a profound impact on the central nervous system. Given the similarity between the necrosis of neurons in the spinal cord and brainstem observed in a mouse model of enterovirus infection and the distribution of antigens in the central nervous system as seen in humans (Yu et al., 2014), the findings from this study provide robust support for further investigation into the impact of CV-A6 infection on the human central nervous system. Additionally, miRNA dysregulation can potentially impair the integrity of the blood-brain barrier, trigger oxidative stress, exacerbate inflammatory responses, and ultimately contribute to a range of central nervous system disorders (Li et al., 2020; Wu et al., 2020). It should be noted that after infection, miRNAs participate in various processes and form a network synergy that ultimately results in brain damage and manifestations through multiple pathways. For instance, viral encephalitis can lead to pulmonary and cardiovascular decompensation by damaging the medullary vascular motor center and causing inflammation in the central nervous system (Yu et al., 2014). MIR-143-3p, which is associated with cardiovascular disease, has been found to be significantly upregulated in human myocardial tissues within the infarcted area after myocardial infarction. Additionally, MIR-143-3p and SPRY3 jointly regulate biological functions by activating p38, ERK, and JNK pathways (Huang et al., 2023). In this study, we observed a significant upregulation of miR-143-3P expression in mice on both day 2 and day 4 following CV-A6 infection.

MiR-363-3p is crucial in cerebral ischemia/reperfusion (I/R) injury, mitigating OGD/R-induced cell damage through the reduction of apoptosis and inflammation (Wang et al., 2022). miR-144-3p shows a negative correlation with IL-1β, and miR-144-3p mimics have the ability to downregulate IL-1β levels (Lin et al., 2021). MiR-19a-3p can suppress the expression of Wnt1, β-catenin, cyclin D1, and c-Myc, specifically targeting Wnt1 via the Wnt/β-catenin signaling pathway to suppress cell proliferation (Wei et al., 2021). Additionally, experimental evidence suggests that miR-709 can activate the Wnt/β-catenin signaling pathway by targeting GSK3β (Chen et al., 2014). The expression of miR-363-3p, miR-144-3p, miR-19a-3p and miR-709 were significantly down- regulated at both 2 dpi and 4dpi in this study. In conclusion, this study identified a significant number of miRNAs with differential expression associated with the central nervous system.

According to the GO enrichment analysis, the ranking terms for differentially expressed miRNAs include “transcription, DNA−templated”, “nervous system development”, “positive regulation of transcription, DNA−templated”, “synapse”, “cell junction”, “cell projection” and “protein/DNA/ATP binding”. These terms suggest that miRNAs play a crucial role in regulating RNA transcription, leading to significant differential expression of nervous system function. The miRNA molecule binds to the 3’ untranslated region (3’UTR) of target mRNA, thereby impeding mRNA translation or suppressing transcription initiation by inducing mRNA degradation. This regulatory mechanism effectively modulates protein expression levels and plays a pivotal role in governing cellular processes such as proliferation, differentiation, development, apoptosis, and other biological phenomena (O'Brien et al., 2018). MiR-3473b can regulate the secretion of pro-inflammatory mediators by targeting TREM2/ULK1 expression, thereby modulating the role of autophagy in the pathogenesis of Parkinson’s disease (PD) inflammation (Lv et al., 2021). In a study exploring the mechanisms of enterovirus EV71 infection, it was discovered that in mice infected with EV71, miR-342-5p can inhibit the expression of CTNNBIP1 by targeting its 3’ UTR region and reducing its binding to CTNNB1, thereby enhancing the interaction between CTNNB1 and TCF4. This intensification activated the Wnt signaling pathway-mediated type I interferon response in nerve cells and tissues, and diminished the antiviral innate immune response through the Wnt/CTNNB1 signaling pathway (Tang et al., 2023). In our sequencing results, miR-3473b and miR-342-5p exhibited similar trends.

Additionally, based on the outcomes of KEGG enrichment analysis, the predominant dysregulated signaling pathways observed in 2dpi and 4dpi mice include but are not limited to the Wnt signaling pathway, Notch signaling pathway, and phosphatidylinositol signaling pathway.

The Wnt signaling pathway plays a crucial role in the regulation of downstream gene expression within the nervous system, encompassing axon guidance during neural development, axon regeneration following nerve injury, metabolic patterns of nerve cells, and oxidative stress modulation in nerve cells (Kostes and Brafman, 2023). Within the central nervous system, Wnt signaling stimulates neurogenesis in the adult hippocampus, facilitates the formation of synapses, elevates neuronal discharge activity, and bolsters neuronal plasticity and neurotransmission (Farías et al., 2010; Oliva and Inestrosa, 2015). Wnt signaling aids in the repair of blood vessels in the central nervous system and the development of the blood-brain barrier and share communications with microglial (Hübner et al., 2018; Van Steenwinckel et al., 2019). Abnormalities in the Wnt signaling pathway are associated with the development of chronic inflammatory diseases such as neuroinflammation, cancer inflammation, and metabolic inflammation. Microglia serve as the primary regulators of neuroinflammation. Activation of the Wnt signaling pathway can trigger microglia, setting them in a pro-inflammatory state (Yang and Zhang, 2020), while simultaneously accelerating neurodegeneration via immune and inflammatory response (Davis and Pennypacker, 2017; Gibson et al., 2019). In this study, similar to many viral infections (Hepatitis B Virus, Hepatitis C virus, and herpes simplex virus 1) significant upregulation of the Wnt signaling pathway was observed in mice infected with CV-A6 at 2dpi and 4dpi, accompanied by notable disruption of multiple miRNAs within the pathway (Yin et al., 2017; Street et al., 2005; Milward et al., 2010; Zhu and Jones, 2018). We further validated several key proteins involved in this pathway and confirmed their expression levels, including upregulation of β-Catenin and Cyclin D1-C terminal protein expression, and downregulation of LEF1 and Axin2 protein expression levels. These findings imply a plausible link between upregulation of the Wnt signaling pathway and central nervous system injury induced by CV-A6 infection. These findings imply that the central nervous system damage caused by CV-A6 infection may be associated with a disruption in the Wnt signaling pathway, likely resulting from the activation and pro-inflammatory state induced by the Wnt signaling pathway acting on its primary target, microglia. Naturally, the precise mechanism of action still requires further investigation.

The Notch signaling pathway is expressed in neural stem cells during development and plays a crucial role in regulating the determination of glial cell fate (Mora and Chapouly, 2023). After binding to the receptor, Notch ligands (such as Delta-like 1 (DLL1)) can initiate the Notch signaling pathway, thereby triggering the release of the intracellular domain of the receptor (NICD) into the nucleus. In the nucleus, NICD interacts with effector binding proteins and transcription activators to finely regulate protein expression (Yin et al., 2024). The Notch and Wnt signaling pathways intersect at numerous points, mutually interfering in diverse systems. The outcomes of this crosstalk are somewhat contentious and may encompass diverse components in distinct experimental systems (Espinosa et al., 2003). β-catenin binds to the intracellular domain of Notch1 to upregulate the expression of Notch signaling pathway and inhibit neuronal differentiation (Shimizu et al., 2008). The Notch signaling pathway enhances the expression of Lgr5 (a Wnt signaling pathway agonist receptor) and elevates the cell count in spinal cord motor neurons (Mußmann et al., 2014). During the development of the mouse auditory plate, the Wnt signaling pathway modulates the Notch ligand Jagged1 (Jayasena et al., 2008). Our findings demonstrate a significant up-regulation of both the Wnt and Notch signaling pathways in mouse brain tissues at 2dpi and 4dpi, suggesting a potential synergistic interplay between these two pathways within the central nervous system.

Phospholipase C (PLC) plays a key role in the phosphatidylinositol signaling system and is the core of many important interlocking regulatory networks (Berry et al., 2016). The deregulation of PLC signaling is associated with many brain diseases such as Alzheimer’s Disease (AD), Huntington’s Disease (HD), Epilepsy, Schizophrenia, Bipolar disorder, and Depression (Yang et al., 2016). Numerous studies indicate that PLC-g1(PLC-γ1);is implicated in neurotrophic factor signaling pathways and diverse neuronal activities, including neurite growth, neuronal cell migration, and synaptic plasticity. PLC-g1(PLC-γ1);may play a role in neuronal function and associated brain disorders (Jang et al., 2013). PLC-g1 is associated with tau protein in human neuroblastoma cells (Jenkins and Johnson, 1998). Tau interacts with the PLC-g1 SH3 domain through its PXXP motif (Reynolds et al., 2008). According to the postulated mechanism of AD, hyperphosphorylated tau and neurofibrillary tangles (Mudher and Ovestone, 2002) ultimately lead to the breakdown of the neuronal transport system and neuronal death via microtubule disassembly (Iqbal et al., 2005). Consistent with this, PLC-g1 expression in cortical tissue of AD patients is significantly reduced compared to the control group (Shimohama et al., 1995). In this study, we observed a significant decrease in the expression of PLCG1 in mice infected with CV-A6, suggesting that the phosphatidylinositol signaling system may be involved in the damage of the central nervous system caused by CV-A6.

In summary, this study unveiled alterations in the miRNA spectrum within brain tissues of CV-A6-infected mice and utilized bioinformatics technology to predict the structure and function of these miRNAs. Incorporating bioinformatics analysis, this study proposes that the brain tissue damage, resulting from CV-A6 infection in mice, may be associated with the activation and pro-inflammatory state of microglia, triggering a series of reactions, neuronal necrosis, synaptic disruption, and blood-brain barrier damage, leading to brain injury. It is important to note that these conclusions are primarily based on bioinformatics predictions; further research necessitates additional experimental data support. Moreover, further research is required to investigate the alterations and interrelationships between glial cells and neurons in mice following CV-A6 infection. The analysis revealed potential associations between central nervous system injury and dysfunctions in Wnt signaling pathway, Notch signaling pathway, as well as phosphatidylinositol signaling pathway – areas that will be explored further in our future investigations.

## Data Availability

The datasets presented in this study can be found in online repositories. The names of the repository/repositories and accession number(s) can be found in the article/**Supplementary Material**.
