# Supplementary figures and images for: Analysis of miRNAs involved in mouse brain injury upon Coxsackievirus A6 infection
Source: Front Cell Infect Microbiol. 2024 Aug 22;14:1405689. doi: 10.3389/fcimb.2024.1405689 (PMC11374775; doi:10.3389/fcimb.2024.1405689)

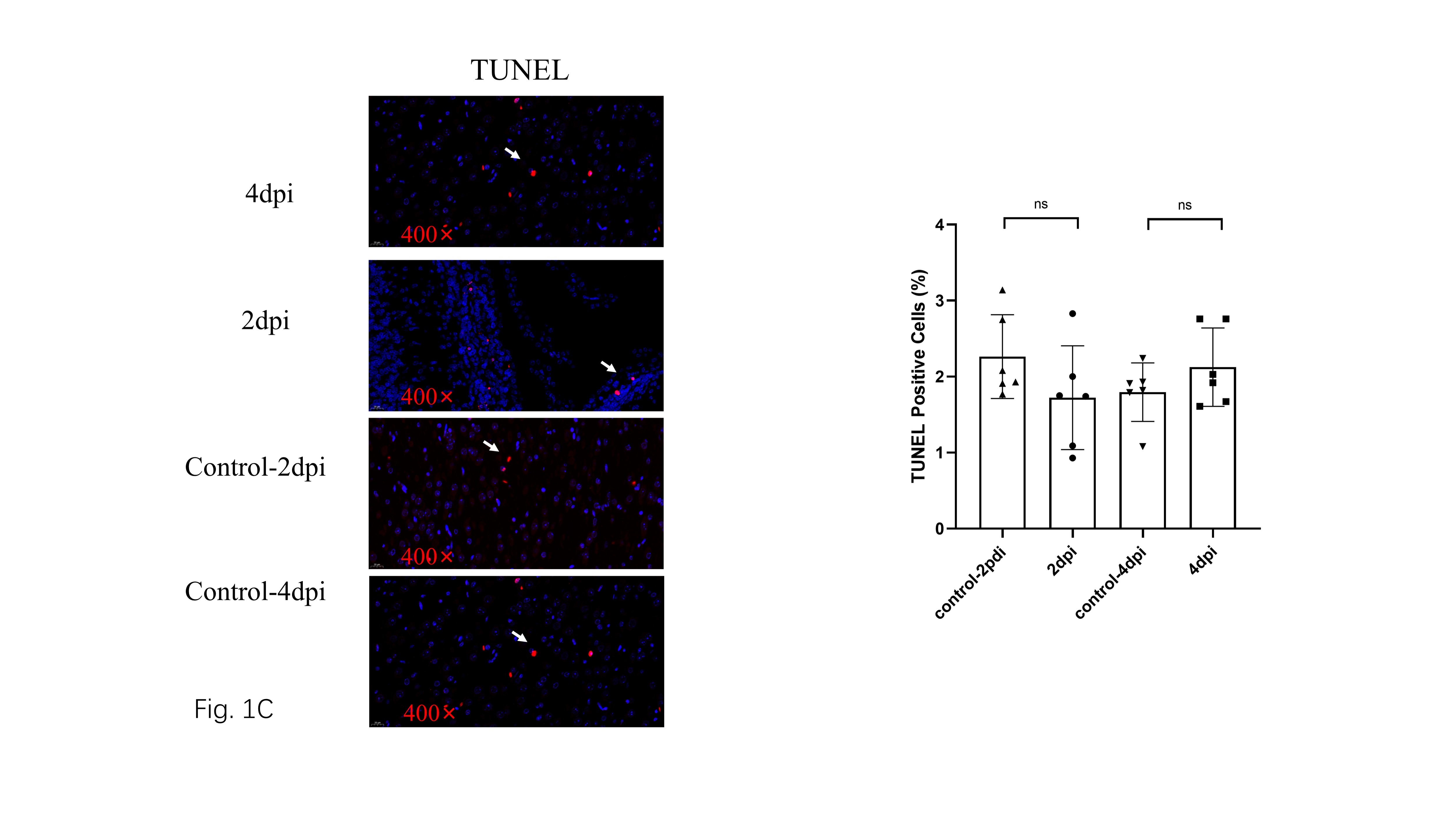

Supplement: Supplementary file 1 [file Image1.jpeg]

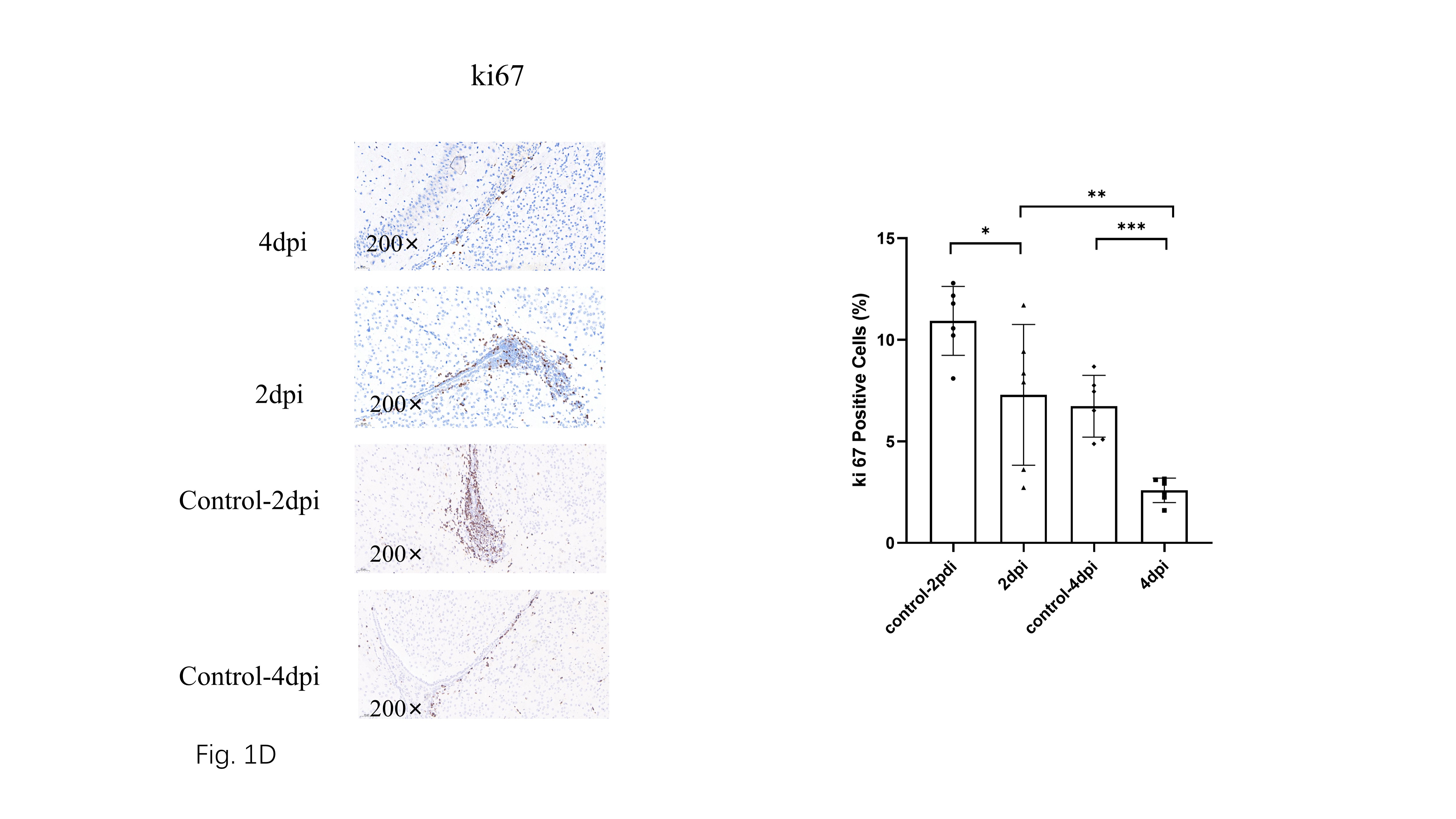

Supplement: Supplementary file 2 [file Image2.jpeg]
