# Supplementary material for: Analysis of miRNAs involved in mouse brain injury upon Coxsackievirus A6 infection
Source: Front Cell Infect Microbiol. 2024 Aug 22;14:1405689. doi: 10.3389/fcimb.2024.1405689 (PMC11374775; doi:10.3389/fcimb.2024.1405689)
Supplement: Supplementary file 3 [file Table1.docx]

Table S1: The differential co-expressed miRNAs.

| miRNA | 2dpi （log2FoldChange） | 4dpi （log2FoldChange） | 4dpi vs 2dpi（log2FoldChange） |
| --- | --- | --- | --- |
| mmu-miR-344g-3p | 5.363716 | 5.5859 | —— |
| mmu-miR-7080-3p | 4.280975 | 5.0412 | —— |
| mmu-miR-466i-3p | 5.176019 | 5.0241 | —— |
| mmu-miR-29a-5p | 4.490641 | 4.8466 | —— |
| mmu-miR-3081-3p | 5.162123 | 4.6952 | —— |
| mmu-miR-3085-5p | 4.27843 | 4.6367 | —— |
| mmu-miR-376c-5p | 3.667759 | 4.3854 | —— |
| mmu-miR-669f-5p | 3.937808 | 4.3751 | —— |
| mmu-miR-489-3p | 4.079681 | 4.2940 | —— |
| mmu-miR-7047-3p | 4.075561 | 4.2660 | —— |
| mmu-miR-219a-1-3p | 4.525457 | 4.0049 | —— |
| mmu-miR-487b-5p | 3.65296 | 3.9136 | —— |
| mmu-miR-449c-5p | 5.628969 | 3.8956 | -1.7739466 |
| mmu-novel-129 | 3.52193 | 3.5843 | -3.1603975 |
| mmu-miR-6977-3p | 2.954415 | 3.2821 | —— |
| mmu-novel-82 | 2.806731 | 3.2656 | —— |
| mmu-miR-702-5p | 4.393391 | 3.2223 | -1.2202232 |
| mmu-miR-429-3p | 4.948741 | 2.9750 | -2.0180791 |
| mmu-miR-200a-3p | 4.924223 | 2.9300 | -2.038442 |
| mmu-novel-59 | 2.950081 | 2.9263 | —— |
| mmu-miR-200b-3p | 4.49835 | 2.9151 | —— |
| mmu-miR-653-5p | 2.618774 | 2.8967 | —— |
| mmu-miR-491-3p | 2.45826 | 2.7109 | —— |
| mmu-novel-40 | 2.806137 | 2.6952 | —— |
| mmu-novel-17 | 2.466172 | 2.6038 | 3.66162994 |
| mmu-miR-582-3p | 2.141084 | 2.5414 | —— |
| mmu-miR-669d-5p | 2.545392 | 2.4992 | —— |
| mmu-miR-329-5p | 1.8932 | 2.4766 | —— |
| mmu-miR-6540-3p | 2.338751 | 2.3989 | —— |
| mmu-miR-673-5p | 2.222804 | 2.3573 | —— |
| mmu-miR-27a-5p | 2.249472 | 2.3088 | —— |
| mmu-miR-135b-3p | 2.806755 | 2.3087 | —— |
| mmu-miR-669b-5p | 2.658619 | 2.1029 | —— |
| mmu-miR-467a-3p | 1.263803 | 1.9600 | —— |
| mmu-miR-467d-3p | 1.263803 | 1.9600 | —— |
| mmu-miR-467d-5p | 1.742639 | 1.9567 | —— |
| mmu-miR-483-3p | 2.239472 | 1.9265 | —— |
| mmu-miR-351-3p | 2.100326 | 1.6815 | —— |
| mmu-miR-344b-3p | 1.052453 | 1.6078 | —— |
| mmu-miR-466a-3p | 1.324972 | 1.5872 | —— |
| mmu-miR-466b-3p | 1.324972 | 1.5872 | —— |
| mmu-miR-466c-3p | 1.324972 | 1.5872 | —— |
| mmu-miR-466e-3p | 1.324972 | 1.5872 | —— |
| mmu-miR-466p-3p | 1.324972 | 1.5872 | —— |
| mmu-miR-300-5p | 1.45066 | 1.4814 | —— |
| mmu-miR-27b-3p | 1.16632 | 1.4546 | —— |
| mmu-miR-326-3p | 1.374737 | 1.4286 | —— |
| mmu-miR-34b-5p | 1.29631 | 1.4248 | —— |
| mmu-miR-345-3p | 1.436468 | 1.4183 | —— |
| mmu-miR-674-5p | 1.178539 | 1.4115 | —— |
| mmu-miR-26a-1-3p | 2.501246 | 1.4035 | —— |
| mmu-miR-143-3p | 1.79073 | 1.3711 | —— |
| mmu-miR-344-3p | 1.284125 | 1.3589 | —— |
| mmu-miR-3059-5p | 1.090676 | 1.3293 | —— |
| mmu-miR-140-5p | 1.552584 | 1.3271 | —— |
| mmu-miR-30c-2-3p | 1.215819 | 1.3031 | —— |
| mmu-miR-344f-5p | 1.368888 | 1.2427 | —— |
| mmu-miR-410-3p | 1.247111 | 1.2303 | —— |
| mmu-miR-1249-3p | 1.089954 | 1.2283 | —— |
| mmu-let-7i-3p | 1.271078 | 1.1336 | —— |
| mmu-miR-34c-5p | 1.173429 | 1.1227 | —— |
| mmu-miR-145a-5p | 1.521011 | 1.1022 | —— |
| mmu-miR-296-5p | 1.01423 | 1.0832 | —— |
| mmu-miR-434-5p | 1.000552 | 1.0556 | —— |
| mmu-miR-540-3p | 1.052561 | 1.0550 | —— |
| mmu-miR-125a-3p | 1.06942 | 1.0275 | —— |
| mmu-miR-22-3p | -1.29674 | -1.0053 | —— |
| mmu-miR-501-3p | -1.13673 | -1.1180 | —— |
| mmu-miR-98-3p | -1.11584 | -1.1324 | —— |
| mmu-miR-214-3p | 1.633126 | -1.1525 | -2.8422151 |
| mmu-miR-652-3p | -1.12735 | -1.1867 | —— |
| mmu-miR-191-5p | -1.40529 | -1.2750 | —— |
| mmu-miR-192-5p | -1.45082 | -1.3361 | —— |
| mmu-miR-194-5p | -1.32532 | -1.4457 | —— |
| mmu-miR-547-3p | -1.35133 | -1.5007 | —— |
| mmu-miR-186-5p | -1.60754 | -1.5596 | —— |
| mmu-miR-505-5p | -1.47663 | -1.5761 | —— |
| mmu-miR-15a-5p | -1.40843 | -1.8749 | —— |
| mmu-novel-136 | -1.78172 | -2.1066 | —— |
| mmu-miR-19b-3p | -1.55726 | -2.1264 | —— |
| mmu-miR-21a-5p | -1.82391 | -2.1714 | —— |
| mmu-miR-15b-3p | -1.64436 | -2.1825 | —— |
| mmu-miR-425-5p | -2.2824 | -2.2599 | —— |
| mmu-miR-19a-3p | -1.22011 | -2.2836 | -1.0925489 |
| mmu-miR-18a-5p | -1.46469 | -2.3345 | —— |
| mmu-miR-16-5p | -2.15204 | -2.3856 | —— |
| mmu-miR-142a-5p | -1.86155 | -2.3918 | —— |
| mmu-miR-320-3p | -2.26833 | -2.3948 | —— |
| mmu-novel-5 | -1.8303 | -2.4486 | —— |
| mmu-miR-92a-3p | -2.07542 | -2.6567 | —— |
| mmu-miR-93-5p | -2.08485 | -2.7037 | —— |
| mmu-miR-500-3p | -3.05736 | -2.9283 | —— |
| mmu-miR-106b-5p | -2.83977 | -3.4343 | —— |
| mmu-miR-223-3p | -2.58259 | -3.4435 | —— |
| mmu-miR-185-5p | -4.20125 | -3.7011 | —— |
| mmu-novel-3 | -1.32687 | -3.8333 | -2.9087753 |
| mmu-miR-106b-3p | -3.47157 | -3.9839 | —— |
| mmu-miR-25-3p | -3.37525 | -4.0640 | —— |
| mmu-miR-96-5p | -2.35362 | -4.0682 | -1.7376833 |
| mmu-miR-1957a | -4.65562 | -4.1811 | —— |
| mmu-miR-18a-3p | -4.17422 | -4.3565 | —— |
| mmu-miR-690 | -1.8275 | -4.3777 | -2.5716285 |
| mmu-miR-5128 | -2.60657 | -4.5900 | —— |
| mmu-miR-5100 | -1.29008 | -4.5930 | -3.3565739 |
| mmu-novel-144 | -2.97105 | -4.6423 | —— |
| mmu-miR-423-5p | -4.58708 | -4.6951 | —— |
| mmu-miR-1195 | -3.69981 | -4.7962 | —— |
| mmu-miR-20b-5p | -2.67184 | -4.8582 | -2.2076695 |
| mmu-novel-119 | -2.2832 | -5.4164 | —— |
| mmu-miR-144-3p | -3.40204 | -5.4664 | -2.0880532 |
| mmu-miR-3963 | -2.84102 | -5.6673 | -2.8607507 |
| mmu-miR-486a-5p | -4.81446 | -5.7186 | —— |
| mmu-miR-486b-5p | -4.81446 | -5.7186 | —— |
| mmu-novel-1 | -2.88124 | -5.7559 | —— |
| mmu-novel-151 | -3.28641 | -5.8135 | —— |
| mmu-miR-451a | -5.76578 | -6.4601 | —— |
| mmu-miR-363-3p | -3.10141 | -6.6093 | -3.5308234 |
| mmu-miR-709 | -6.00493 | -6.7638 | —— |
| mmu-novel-36 | -7.93701 | -7.2651 | —— |
| mmu-novel-115 | -6.68625 | -7.4718 | —— |
| mmu-miR-192-3p | -6.70532 | -7.4906 | —— |
| mmu-novel-37 | -7.0152 | -7.7977 | —— |
| mmu-novel-98 | -2.77006 | -8.0901 | —— |
| mmu-novel-130 | -7.95771 | -8.7097 | —— |
| mmu-novel-41 | -5.9782 | -9.5250 | —— |
| mmu-miR-3473e | -9.6725 | -10.4262 | —— |
| mmu-miR-3473b | -9.68921 | -10.4429 | —— |
| mmu-novel-91 | -7.0694 | -22.8899 | —— |
